# Advances in automatic identification of flying insects using optical sensors and machine learning

**DOI:** 10.1038/s41598-021-81005-0

**Published:** 2021-01-15

**Authors:** Carsten Kirkeby, Klas Rydhmer, Samantha M. Cook, Alfred Strand, Martin T. Torrance, Jennifer L. Swain, Jord Prangsma, Andreas Johnen, Mikkel Jensen, Mikkel Brydegaard, Kaare Græsbøll

**Affiliations:** 1 Section for Animal Welfare and Disease Control, Department of Veterinary and Animal Sciences, Faculty of Health and Medical Sciences, University of Copenhagen, 1870 Frederiksberg, Denmark; 2 FaunaPhotonics APS, Ole Maaløes Vej 3, 2200 Copenhagen N, Denmark; 3 Department of Biointeractions and Crop Protection, Rothamsted Research, Harpenden, UK; 4 Xarvio Digital Farming Solutions, BASF Digital Farming GmbH, Albrecht-Thaer-Strasse 34, Münster, Germany; 5 Lund Laser Centre, Department of Physics, Lund University, Sölvegatan 14, 223 62 Lund, Sweden; 6 DTU Compute, Technical University of Denmark, 2800 Kongens Lyngby, Denmark

**Keywords:** Agroecology, Machine learning, Entomology

## Abstract

Worldwide, farmers use insecticides to prevent crop damage caused by insect pests, while they also rely on insect pollinators to enhance crop yield and other insect as natural enemies of pests. In order to target pesticides to pests only, farmers must know exactly where and when pests and beneficial insects are present in the field. A promising solution to this problem could be optical sensors combined with machine learning. We obtained around 10,000 records of flying insects found in oilseed rape (*Brassica napus*) crops, using an optical remote sensor and evaluated three different classification methods for the obtained signals, reaching over 80% accuracy. We demonstrate that it is possible to classify insects in flight, making it possible to optimize the application of insecticides in space and time. This will enable a technological leap in precision agriculture, where focus on prudent and environmentally-sensitive use of pesticides is a top priority.

## Introduction

Modern day agriculture entails a delicate balance between increasing crop production to accommodate an increasing population^1–3^ while limiting the use of pesticides in order to reduce the development of resistance to insecticides and to reduce other negative side effects including affecting non-target organisms, environmental pollution and human health issues^4–6^. Additionally, the terrestrial insect population has declined in some areas by more than 75% over the last 27 years^7,8^. This has been linked to the use of pesticides among other factors^9^. Thus, there is a demand for reducing pesticide use, and not least to apply them with as little risk for non-target insects as possible. The key to reach this goal is to optimize the use of pesticides to periods and areas where pests are present and other insects are least affected. This inevitably involves recognition of the insects in the field.

Oilseed rape (OSR) is the third-largest vegetable oil produced worldwide with 68.5 million tonnes produced in 2016^10^. In northern and central Europe alone, 26.3 million tonnes were produced in 2017 over an area of 6.7 million hectares^11^. There are six major pests attacking OSR in this region which means that the crop usually receives 1 to 5 insecticide applications per year^12,13^. We focus on four of the most important pests: cabbage stem flea beetles (*Psylliodes chrysocephala*) the adults of which can cause complete crop failure due to feeding damage during establishment^14^ and the larval mining within plant stems causes considerable damage and yield loss^12,15^; pollen beetles (*Brassicogethes aeneus*) which can reduce yield by 70% through feeding damage if no pesticides are used ^16^; cabbage seed weevils (*Ceutorhynchus obstrictus*) which can reduce the crop yield by up to 18% due to larval feeding on seeds^12^; and Brassica pod midges (*Dasineura brassicae*) the larvae of which cause pod shatter and losses of seed yield by up to 82%^12^.

Monitoring OSR crops for pest abundance to determine whether or not pest population thresholds have been exceeded is an important process in order to optimize application of pesticides, thereby minimizing their use^12^. Plant scouting is traditionally used to achieve this but is time-consuming; yellow water traps or sticky traps are also used^12,17,18^ and although there are accurate web-based decision support systems that can optimise monitoring effort^17,19^, both methods require manual identification of insects. Tools for automatic identification of insects are starting to emerge^20–22^. However, these traps often only reflect the local conditions around the trap, which can be different from the situation in the whole field, as pest populations are not uniform. Precision farming, whereby insecticide applications are adjusted according to measured variability in insect abundance, is not yet possible due to the lack of appropriate sensors and associated imaging technology. Recently, progress has been made in research towards remote monitoring of insects^23,24^. There are many studies of monitoring insect activity using remote sensing methods. For instance, Potamitis et al.^25^ described the augmentation of plastic traps to identify insect species based on wing beat frequencies. Zhu et al.^26^ used lidar remote sensing to detect insect activity in rice fields in China and concluded that extensive collaboration between entomologists and physicists is needed to link the recorded signals to insect species. Genoud et al.^27^ used a lidar system to remotely detect and distinguish between mosquito species based only on their wing beat frequency using a Bayesian approach. Recently, Tauc et al.^28^ have described progress with a pan-and-tilt system able to detect insects in 3D, without identification of insect groups or species. However, such monitoring systems must be validated on known free flying insects, to link the signals to the presence of each species or species group. This is labour-intensive, and has previously only been conducted in a limited number of studies on disease vector insects^29,30^, and insects in a meadow^31^.

The aim of this study was to test a novel prototype optical sensor system for detection of flying insects. As a proof-of-concept, we used machine learning to quantify the detection of OSR pests under controlled laboratory conditions.

## Results

**Figure 1:**
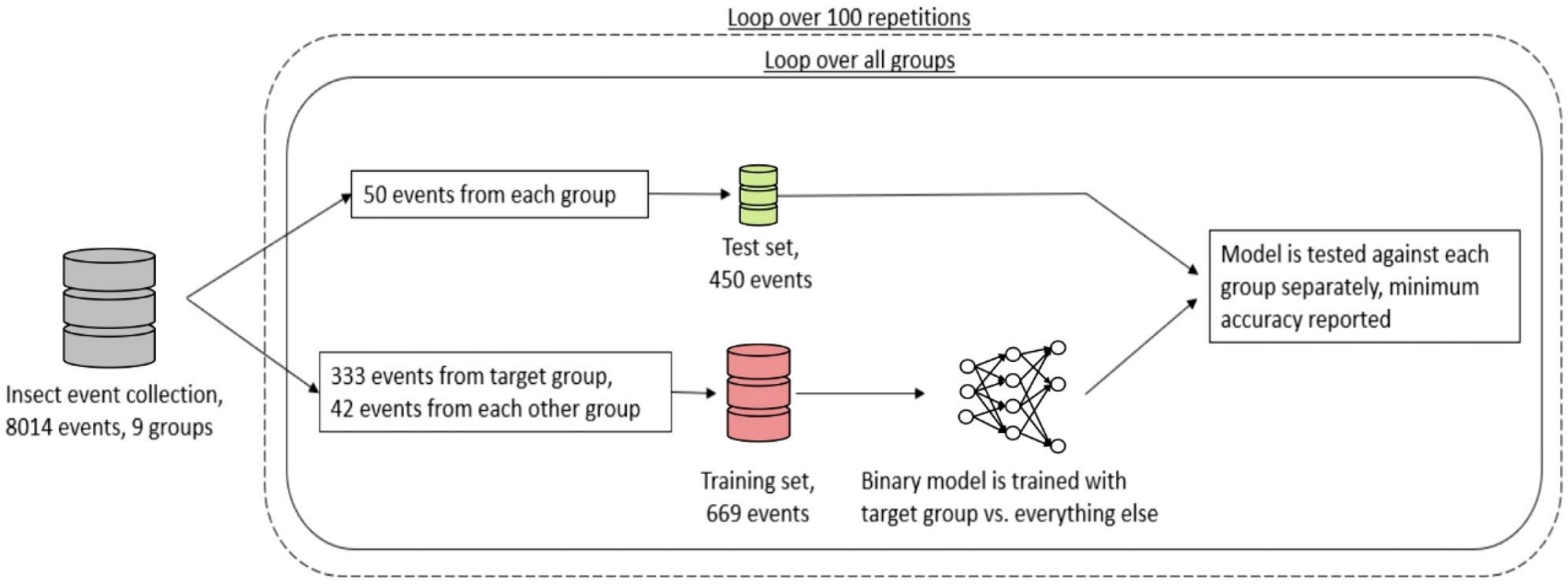
Diagrammatic representation of the process used to classify the insect groups. For each target group, 333 randomly selected target group events are used together with 42 randomly selected events from each of the other groups, to classify the test data consisting of 50 unknown events. The accuracy of the classification is reported. This procedure is repeated in a loop 100 times, to account for stochastic variation.

We tested three different methods to automatically classify the insect groups, splitting the data into a training set and a test set (Fig. 1): first we used the dominant frequency of the Fourier transformation, assuming that this is the wingbeat frequency (WBF) of the insect, for direct comparison between insects (“WBF method”). This assumption is correct for some cases, but could be wrong in some cases where the first harmonic is strongest. In the “Features method” we used a Random Forest classifier to classify the groups^32^. In this method, several other features of the signal were identified including second and third harmonics of the WBF and various ratios between the signals^29,32,33^ (see Supplementary Table 1) and used for classification in decision tree models. Thirdly, we fed the power spectra into a 3-layer Neural Network with rectifier (ReLU) activation functions^34^ implemented in TensorFlow^35^ (“NN method”). For all methods, we used a one-vs-all approach, using a binary classifier classifying the events as “target” or “other”.

### Accuracy

**Figure 2:**
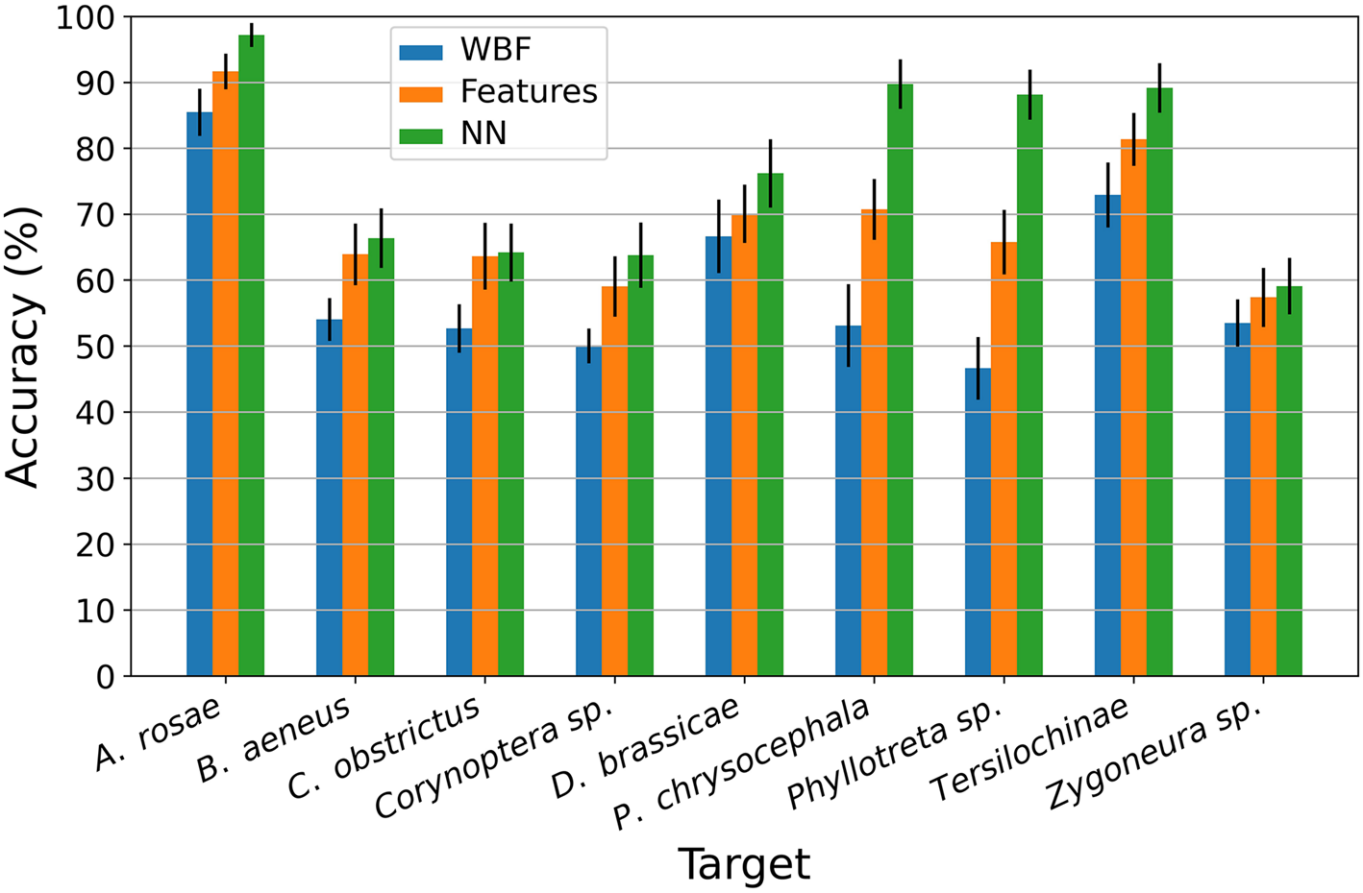
Median classification accuracy (y axis) of one target insect species (x axis) against the most difficult other species (that yielded the lowest average accuracy of 100 repetitions with randomly selected records). Thus, these are the lowest accuracies possible in this study. The error bars show the 90% bootstrapping interval.

The accuracy of classification in the machine learning depends on the composition of the tested population. In this study, sawflies (Hymenoptera) were easily distinguished from pod midges (Diptera), but it was more difficult to differentiate pod midges (Diptera: Cecidomyiidae) from other midge species (Sciaridae: *Corynoptera* sp. and *Zygoneura* sp.). Therefore, training was first conducted on all insect observations, and then accuracy was obtained by classifying one group at a time. The reported accuracy, or the fraction of correctly classified events, is defined as the sum of true positives and true negative divided by the number of events (Fig. 2). The presented result is the average of 100 randomized cross validation folds with random selection of 669 training data points, 333 targets + 336 others (42 of each the other eight species). The test set consisted of 450 data points, (50 per species) randomly selected in each fold.

### Performance

**Figure 3:**
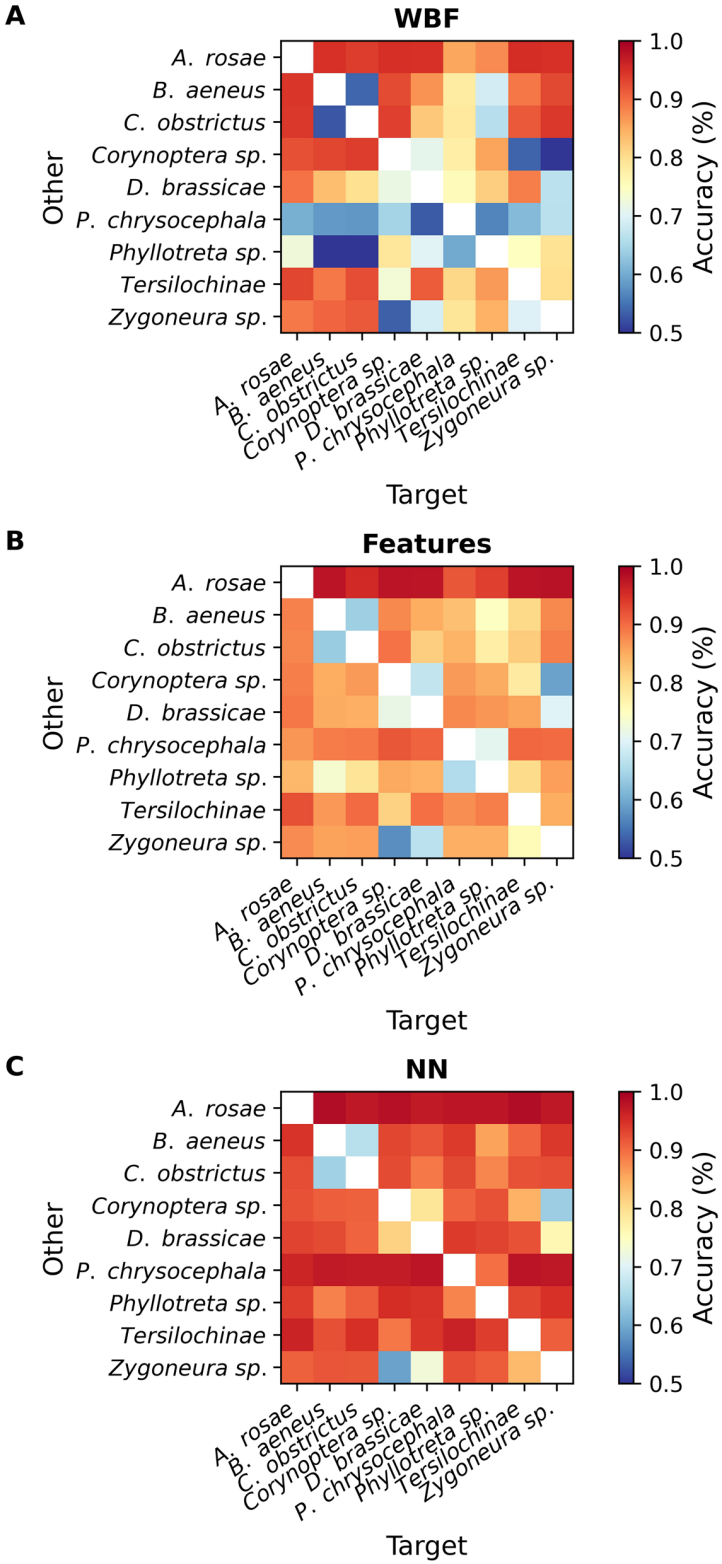
(**A**) Intensity plot of the average accuracy of the classification algorithms using one species as a target and training against a mix of the other species, then testing against specific other species using wing beat frequency as predictor. (**B**) Intensity plot of the average accuracy using one species as target and training against a mix of other species, then testing against specific other species. Here using extracted features as predictors. (**C**) Intensity plot of the average accuracy using one species as target and training against a mix of other species, then testing against specific other species. Here using a neural network (NN) to predict species.

In Fig. 2, we present the lowest accuracy we reached when classifying each insect group against the other groups, one at a time. Of the three methods tested, the NN method performed best when comparing one target species with all other species and also when selecting the species with poorest accuracy (Fig. 2), reaching up to 98% median in accuracy with sawflies as the target species. The relative accuracies between the insect groups are presented in Fig. 3. The Features method was second best in all comparisons, and the WBF method had the lowest accuracy in all comparisons. Specifically, the 90% bootstrapping interval (BI) of the NN method was above the 90% BI of the other two methods for two species (Fig. 2). The NN method had 90% BI interval above the median in four species compared to the Features method, and in seven species compared to the WBF method. For comparison of specific species, the NN method had a higher average accuracy in 70 of 72 comparisons compared to the Features method and 66 out of 72 compared to the WBF method, with the Features method having higher average accuracy in 46 comparisons out of 72 compared to the WBF method (Supplementary Figure 1).

## Discussion

**Figure 4:**
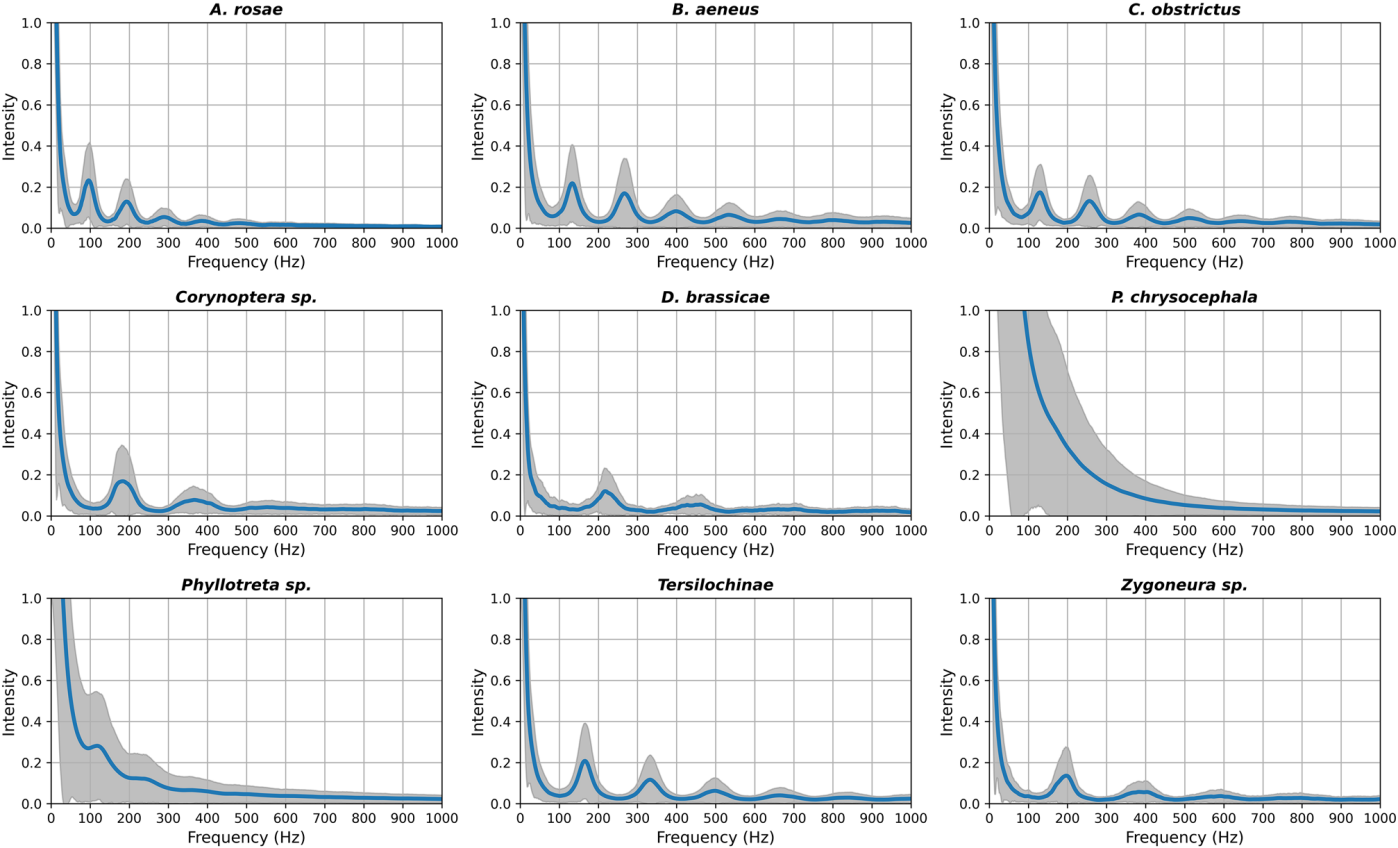
Average spectra of the wing beat frequency signals for the tested insects, showing the harmonics for each classified group. *1H *first harmonic, *2H *second harmonic, etc., exemplified for *B. aeneus*. The grey areas show the interquartile range. The spectra were obtained by taking the average over all four channels (808 nm and 1320 nm, co- and de-polarized signals).

In Fig. 4, we show the WBF harmonics of the studied insects. For each insect, the left peak is the fundamental (first order) harmonic (if present) and for most, the second order harmonic is also clear. Two groups stand out: *Phyllotreta* spp. and *P. chrysocephala* due to their lack of WBF pattern. This is caused by jumping activity rather than flight behaviour. Some of the groups have very similar WBF, which is apparent from the overlapping of dominant frequencies (Fig. 4). This was more pronounced for the related species that have similar physical appearance (e.g. *Corynoptera* sp. and *Zygoneura* sp midges). Therefore, including other features of the Fourier transformed signal made the classification accuracy better. While extracted features sometimes are erroneous, as previously mentioned where the second harmonic in the WBF spectra is chosen rather than the fundamental frequency, the classifier learns the dual distribution of this feature and can still return a valid result. Overall, similarity in appearance between species transferred to lower accuracy across all methods. However, the NN was always better at detecting the groups in single events (Figs. 2 and 3). If the focus is on the insect population rather than identification of single events, an estimate of the fraction of events belonging to a specific species could be made by taking into account the accuracy of the training, which would increase the accuracy on a population level. This will be considered in future work.

These results show that optical signals can be processed using machine learning to distinguish between taxonomic groups of flying insects. This method is a valuable contribution to precision farming because it can help detection of pests faster. Furthermore, it can be applied locally around on the field for rapid identification of local hotspots of pests. In such a situation, the farmer can apply pesticides only where the pests are detected. However, further studies to link *ex-situ* measurements with *in-situ* measurements are needed to validate that the method will work in the field. Combined with other features such as modulation spectra and fluorescence, remote insect recognition will likely be possible in the near future. However, challenges with variation between individual insects, measurement periods and sites will have to be addressed. Furthermore, it will require careful sorting signals and noise in order to develop a fast and reliable system^36^. It will also need to take into account that species without known signatures can be present in the study area. Therefore, a reliable algorithm must be able to quantify the match between the signal and the database records, to measure the quality of the identification. In the future, farmers could utilize the sensor and classification methods to precisely target application of pesticide to areas and periods with confirmed pest activity and little pollinator activity.

## Methods

### Insects

In this study we targeted the analyses on nine species: the four major coleopteran pests of OSR described above plus two additional minor pests—flea beetles *Phyllotreta* spp. and the turnip sawfly *Athalia rosae,* two midge genera *Corynoptera* sp. and *Zygoneura* sp. as common non-target species found in OSR fields and Tersilochine parasitic wasps of the pollen beetle (a mixture of *Tersilochus heterocerus*, *Phradis interstitialis* and *P. morionellus*) as a model natural enemy group. All insects were collected and identified from April to November in an OSR crop on Rothamsted farm (N *51.806146, E 0.359080*), England. The most abundant live insects were collected from a crop of oilseed rape (OSR) on Rothamsted Farm, Hertfordshire, UK using a combination of sweep netting and Malaise trapping^37^ for pollen beetles (*Brassicogethes aeneus*), cabbage seed weevils (*Ceutorhynchus obstrictus*), parasitoids of pollen beetles (*Tersilochinae*), flea beetles (*Phyllotreta* sp.), sawflies (*Athalia rosae*), pod midges (*Dasineura brassicae*) and midges (*Corynoptera* sp. and *Zygoneura* sp.) and electronic aspirators were used to collect cabbage stem flea beetles (*Psylliodes chrysocephala*) at harvest. Collected insects were generally identified live by eye with the exception of midges and parasitoids which were verified after measurements using a binocular microscope. Collected insects were stored in mesh cages with young OSR plants (for cabbage stem flea beetles and flea beetles) or flowering racemes (for cabbage seed weevils and pollen beetles) as food. Pod midges were kept in cages lined with wet sand^38^. Cages were maintained in controlled environment conditions with temperature and Light:Dark regime specific to each insect, until the insects were required for use. Midges, parasitoids and sawflies were generally used on the day of collection whereas the other insects were kept for up to two weeks.

### Measurement procedure

Insects were detected using a prototype optical sensor as shown in Supplementary Figure 1. The sensor transmits a Ø2″ collimated linearly polarized laser beam of both 808 nm and 980 nm, with respective intensity of 1.6 and 3 mW/cm^2^. The backscattered light is collected using a focusing lens, a polarizing beam splitter and two dual-channel sandwich detectors. These constitute four individual channels with co- and de-polarized light recorded separately for both wavelengths. The bandwidth was 5 kHz and sampling frequency was 20 kHz. The measured power spectra are shown in Fig. 4. A similar system is described in more detail in Gebru et al.^29^. Immediately prior to the optical measurements, test insects were housed in small groups in ventilated plastic boxes (18 cm L × 12 cm W × 6 cm D).

Insects were released from the box on a raised platform positioned directly opposite a window (Supplementary Figure 2). The platform was maintained at the same height as the optical sensor ‘beam’ pointed parallel to the window and in front of the release point. The release platform was contained in a Perspex box (60 × 60 × 60 cm) to prevent insects from escaping into the room. A hole was cut into the box at one end to allow the ‘beam’ of the optical sensor into the box and a black panel was fixed onto the Perspex to increase the contrast of the signal (Supplementary Figure 2). The lid was removed, and insects were allowed to fly out. As most insects fly towards the light, the insects flew through the beam towards the window. The flight activity of each batch of insects was recorded for 15 min. Insects were then collected from the Plexiglass box and a fresh batch of insects used. Measurements were made over several days until around 1000 individual traces had been recorded for each group. For each species, we obtained a minimum of 371 records that were used in the analyses.

### Data analysis

Time-windows with individual insect recordings were automatically extracted by thresholding the signal-to-noise-ratio at 10. Peaks above the threshold were eroded and dilated to filter out signal segments that were too short, using the Python *Scipy ndimage* package along the time domain, as described by Jansson^39^. The erosion removes peaks above the threshold shorter than 0.25 ms, remaining peaks are then dilated by 40 ms to cover the initial and final part of the events. The 40 ms corresponds to a frequency resolution of only 50 Hz but the average length of the events used in this study was 85.6 ms corresponding to a frequency resolution of 11.7 Hz. From the extracted events, multiple features were extracted as briefly described in the methods section and according to Gebru et al.^29^. The recorded time signals were also Fourier transformed into the frequency domain and zero padded to 20,000 data points (corresponding to 1 s in the time domain). The final data format, used for classification with the neural network was achieved by down-sampling the absolute values of the Fourier transforms below 1 kHz into 512 points and concatenating the individual channels together into a 1 × 2048 points vector. In the NN method, we used 3-layer neural network with ReLU activation functions implemented in TensorFlow. The layer structure was 2000, 1500, 1000 nodes. The last layer gave a single output with a probability of belonging to the predicted class. We used a cross entropy loss function and optimized the network weights using a standard Adam optimizer (adaptive momentum) and dropout rate of 0.5 on the last layer^40, 41^.

The extracted features for the Features method are listed in Supplementary Table 1. These were fed into the decision tree algorithm^32^. The exact definition of all these features are described in detail in Gebru et al.^29^.

For the training of the different methods, 333 events were randomly selected from each target insect species and 42 events from each of the other eight species (total 336 other insect events). For testing, 50 randomly selected events from the target species and 50 from each other species were selected and a binary test was performed to determine whether events belonged to the target group or not. Training and testing was repeated 100 times for each target species and each method (in total 2700 training sessions and 21,600 test sessions). The composition of the training and test sets were randomized in each fold to cross-validate the results.

Comparison between methods when performing the test session 100 times (bootstrapping) was done by determining whether the mean or median bootstrapped accuracy was within 90% of the bootstrapped accuracy of another method. P-values were not reported because the number of repeats of the bootstrapping decides the power of the comparison^42^; equivalent to us being able to adjust the p-value by adjusting number of bootstraps.

## Supplementary Information

Supplementary Information.

## Data Availability

The datasets used and/or analysed during the current study are available from the corresponding author on reasonable request.
