# Supplementary material for: Advances in automatic identification of flying insects using optical sensors and machine learning
Source: Sci Rep. 2021 Jan 15;11:1555. doi: 10.1038/s41598-021-81005-0 (PMC7810676; doi:10.1038/s41598-021-81005-0)
Supplement: Supplementary file 1 — Supplementary Information. [file 41598_2021_81005_MOESM1_ESM.docx]

Supplementary Information to:

Advances in automatic identification of flying insects using optical sensors and machine learning

Carsten Kirkeby^*1,2^, Klas Rydhmer^2*^, Samantha M. Cook ^3^, Alfred Strand^2^, Martin T. Torrance^3^, Jennifer L. Swain^3^, Jord Prangsma^2^, Andreas Johnen^4^, Mikkel Jensen^2^, Mikkel Brydegaard^2,5^ and Kaare Græsbøll^6^

^*^ These authors contributed equally to this work

^1^Section for Animal Welfare and Disease Control, Department of Veterinary and Animal Sciences, Faculty of Health and Medical Sciences, University of Copenhagen, 1870 Frederiksberg, Denmark

^2^FaunaPhotonics APS, Ole Maaløes Vej 3, DK-2200 Copenhagen N, Denmark.

^3^Department of Biointeractions and Crop protection, Rothamsted Research, Harpenden, UK

^4^Xarvio Digital Farming Solutions, BASF Digital Farming GmbH, Albrecht-Thaer-Strasse 34, Münster, Germany

^5^Lund laser Centre, Department of Physics, Lund University, Sölvegatan 14, SE-223 62 Lund, Sweden.

^6^DTU Compute, Technical University of Denmark, 2800 Kongens Lyngby, Denmark.

Corresponding author: Carsten Kirkeby, ckir@sund.ku.dk

**Supplementary Information**


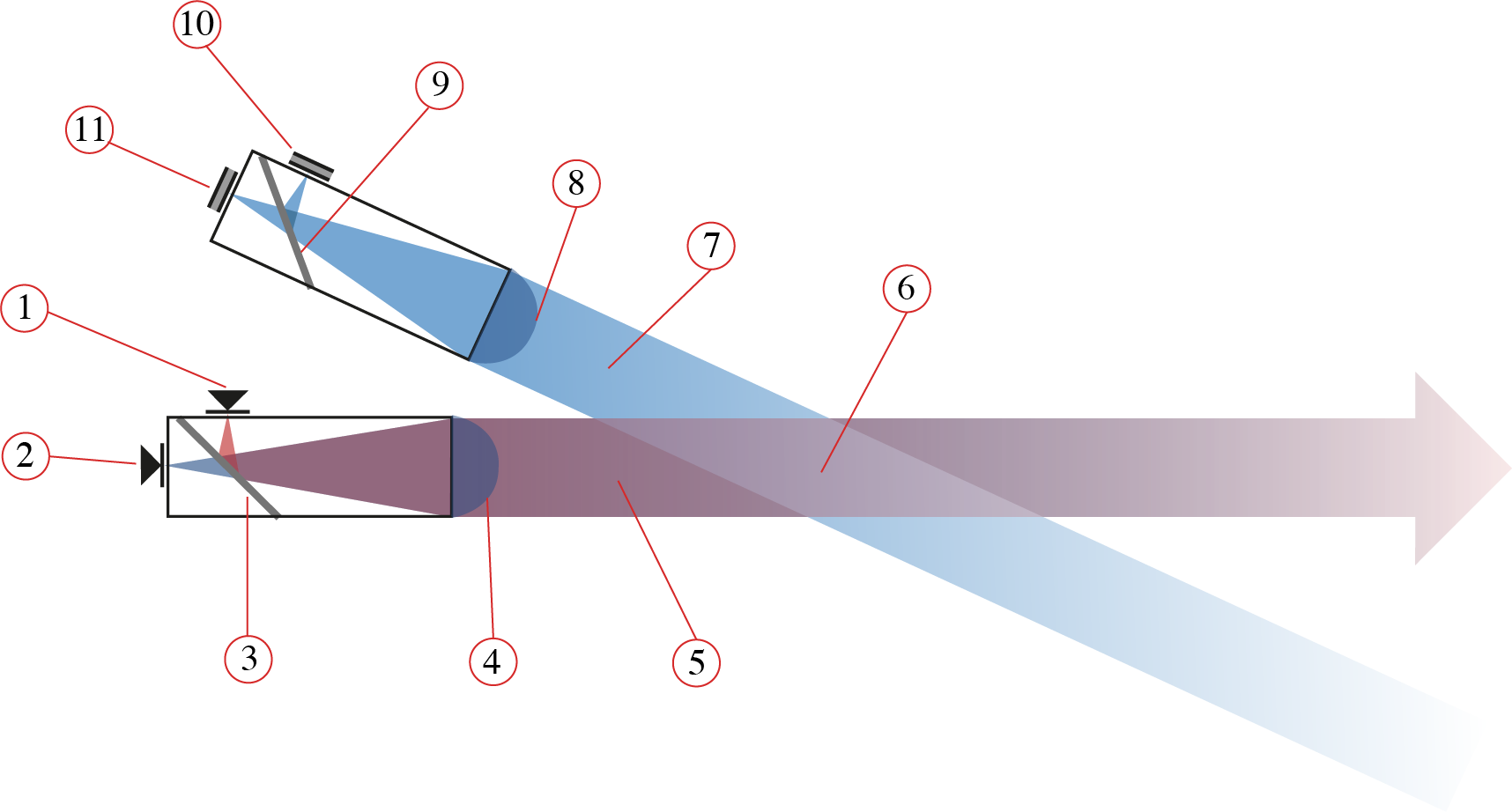


Supplementary Figure 1: Optical sensor setup viewed from above: 1: Laser diode 808 nm, 2: Laser diode 1320 nm, 3: Dichroic mirror, 4: Expander lens, 5: Emitted laser beam, 6: Probe volume, 7: Observed field of view, 8: Focusing lens, 9: Polarizing beam splitter, 10: Sandwich detector (808 nm & 1320 nm, co-polarized signal), 11: Sandwich detector (808 nm & 1320 nm, de-polarized signal). Drawing by Maja Olofsson.


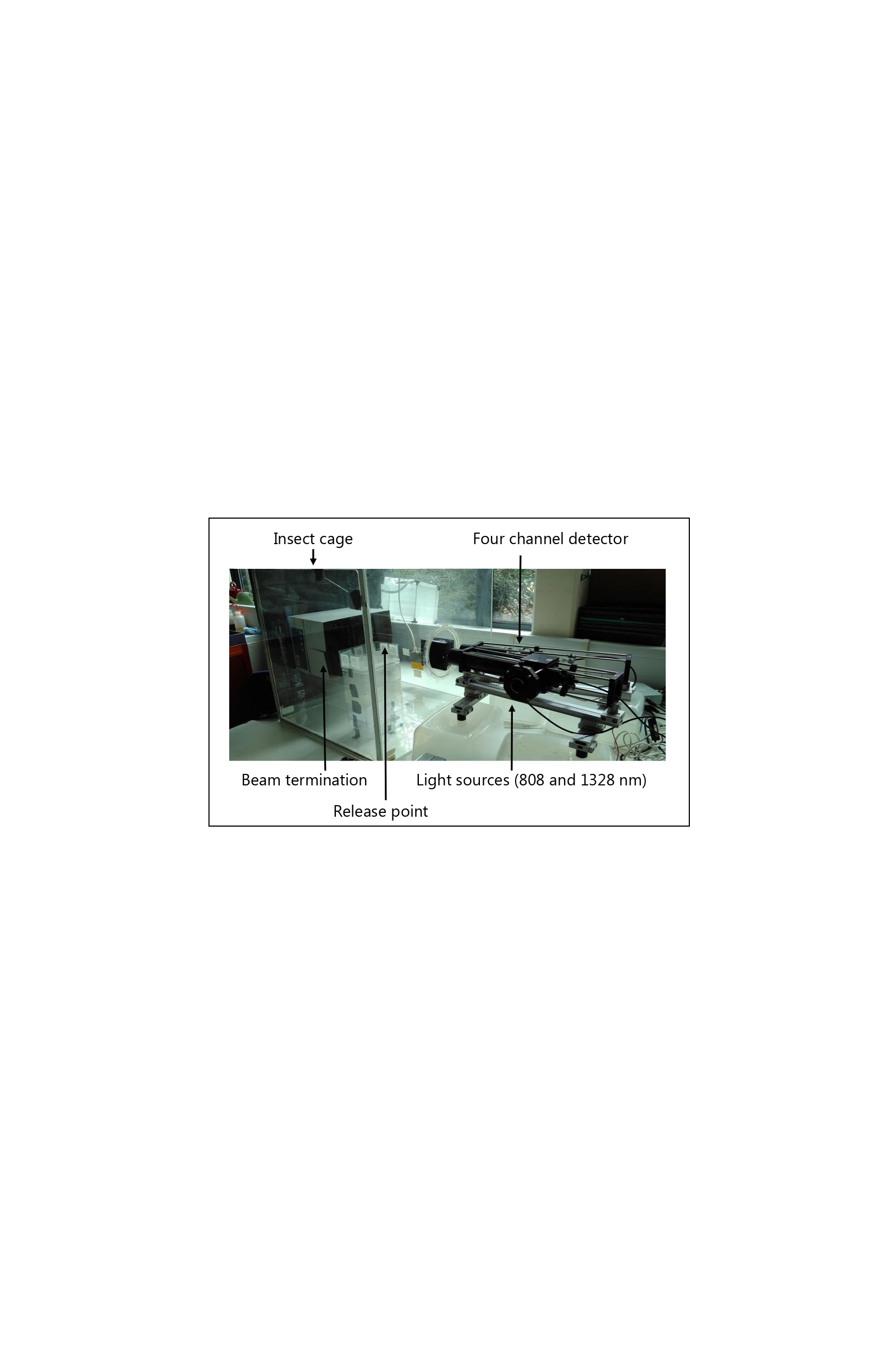


Supplementary Figure 2: Experimental setup of the prototype optical sensor. Photo: Klas Rydhmer.

Supplementary Table 1. The features used in the “Features” method in this study.

| Fundamental frequency |
| --- |
| Frequency of first harmonic |
| Frequency of second harmonic |
| Amplitude of the fundamental frequency in the Fourier transform |
| Amplitude of the first harmonic |
| Amplitude of the second harmonic |
| Full width-half maximum of the fundamental frequency peak in the Fourier transform |
| Full width-half maximum of the first harmonic peak in the Fourier transform |
| Full width-half maximum of the second harmonic peak in the Fourier transform |
| Ratio between amplitude of the fundamental harmonic and the amplitude of the first harmonic, |
| Ratio between amplitude of the fundamental harmonic and the amplitude of the second harmonic, |
| Ratio between amplitude of the first harmonic and the amplitude of the second harmonic, |
| Average amplitude of the body signal, |
| Median amplitude of body signal, |
| Average amplitude of wing signal, |
| Median amplitude of wing signal, |
| Wing mean/wing median, |
| Average of body signal /(body signal  + wing signal), |
| Length of the event |
| Maximum amplitude of event, |
| Average amplitude of the event, and |
| Median amplitude of the event. |
